# Dynamin 2 (DNM2) as Cause of, and Modifier for, Human Neuromuscular Disease

**DOI:** 10.1007/s13311-018-00686-0

**Published:** 2018-11-13

**Authors:** Mo Zhao, Nika Maani, James J. Dowling

**Affiliations:** 1 Genetics and Genome Biology Program, Hospital for Sick Children, Toronto, ON M5G 0A4 Canada; 2 Division of Neurology, Hospital for Sick Children, Toronto, ON M5G 1X8 Canada; 3 Department of Pediatrics, University of Toronto, Toronto, ON M5G 1X8 Canada; 4 Department of Molecular Genetics, University of Toronto, Toronto, ON M5S 1A8 Canada

**Keywords:** Centronuclear myopathy, Charcot–Marie–Tooth neuropathy, DNM2, Congenital neuromuscular disorders, Gene therapy

## Abstract

Dynamin 2 (DNM2) belongs to a family of large GTPases that are well known for mediating membrane fission by oligomerizing at the neck of membrane invaginations. Autosomal dominant mutations in the ubiquitously expressed DNM2 cause 2 discrete neuromuscular diseases: autosomal dominant centronuclear myopathy (ADCNM) and dominant intermediate Charcot–Marie–Tooth neuropathy (CMT). CNM and CMT mutations may affect DNM2 in distinct manners: CNM mutations may cause protein hyperactivity with elevated GTPase and fission activities, while CMT mutations could impair DNM2 lipid binding and activity. DNM2 is also a modifier of the X-linked and autosomal recessive forms of CNM, as DNM2 protein levels are upregulated in animal models and patient muscle samples. Strikingly, reducing DNM2 has been shown to revert muscle phenotypes in preclinical models of CNM. As DNM2 emerges as the key player in CNM pathogenesis, the role(s) of DNM2 in skeletal muscle remains unclear. This review aims to provide insights into potential pathomechanisms related to DNM2-CNM mutations, and discuss exciting outcomes of current and future therapeutic approaches targeting DNM2 hyperactivity.

**Electronic supplementary material:**

The online version of this article (10.1007/s13311-018-00686-0) contains supplementary material, which is available to authorized users.

## Introduction

**Fig. 1:**
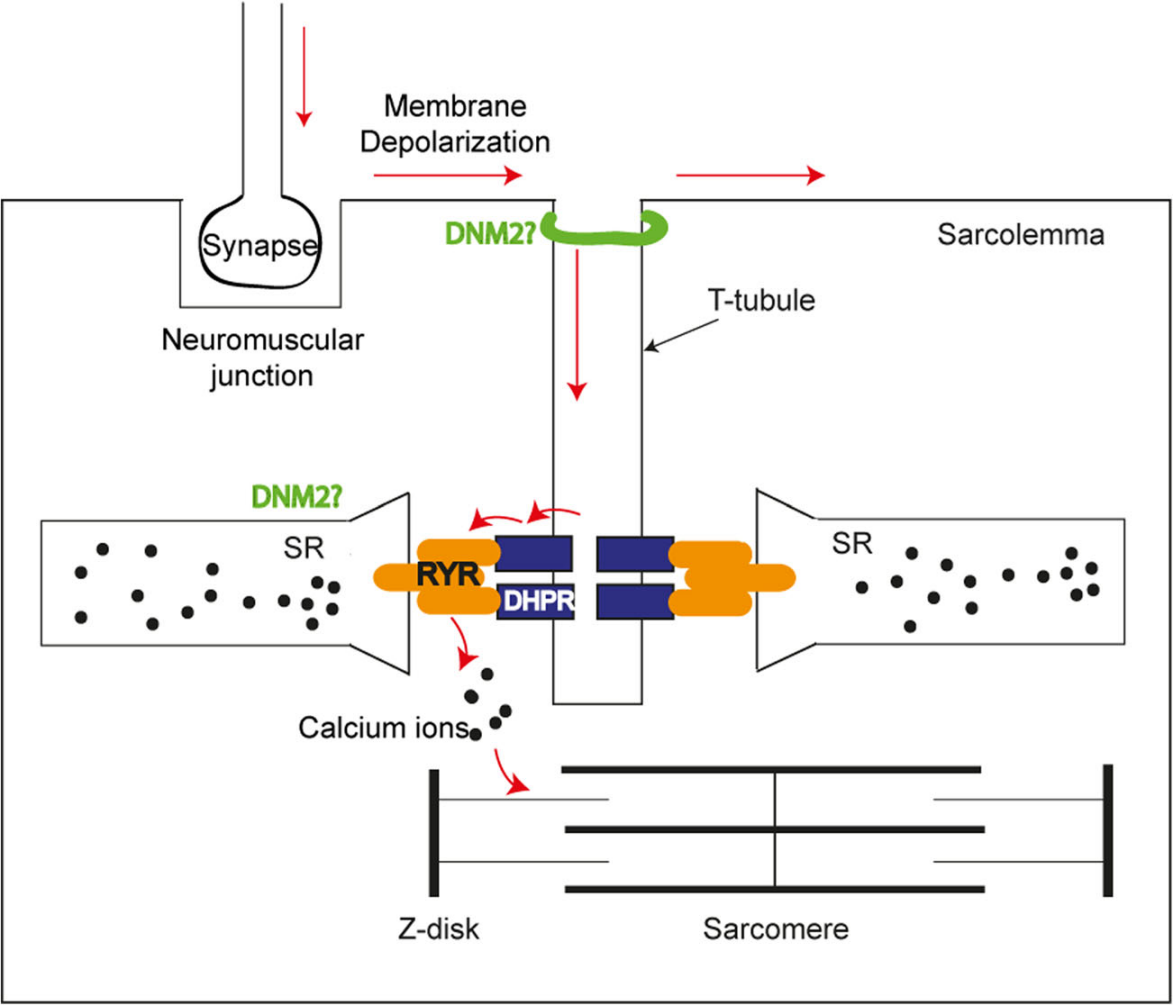
Schematic diagram of the neuromuscular junction, the triad (1 T-tubule and 2 SRs), and the sarcomere during excitation–contraction coupling in skeletal muscle. The nerve impulse arrives at the synapse that transmits and induces membrane depolarization to the sarcolemma and the T-tubules. The DHPR receptor (*blue*) on the T-tubule binds to the Ryr1 receptor (*orange*) on SR membranes, and upon activation triggers Ryr1-regulated release of calcium ions (*black dots*) from the SR. Subsequent binding of calcium ions to the sarcomeric thin filaments triggers sarcomeric contraction. DNM2 is in proximity to the T-tubule (potentially around the neck of the T-tubules) and SR, while its exact subcellular localization remains unclear (DNM2? in *green*). Adobe Illustrator CS6 was used to create this diagram

Mature skeletal muscle utilizes several unique substructures that are dedicated to force production and regulation. These substructures include the neuromuscular junction, the triad, and the sarcomere [1] (Fig. 1). The triad is an essential skeletal muscle substructure formed by the apposition of transverse tubules (T-tubules) and flanking terminal cisternae (enlarged areas of the sarcoplasmic reticulum or SR). T-tubules are a unique tubular membrane system that are continuous with the plasma membrane (sarcolemma) and extend radially into the myocyte interior. The triad primarily acts to regulate/facilitate excitation–contraction (EC) coupling, where T-tubules carry surface depolarization to the junctional contact membrane enriched with voltage sensors (such as the dihydropyridine receptor or DHPR), activating neighboring SR to release internal storage of Ca^2+^ via the ryanodine receptor (RyR1) that further triggers sarcomeric filament sliding for muscle contraction [2]. While much progress has been made in understanding the EC coupling process and the basic contractile unit (i.e., the sarcomere), little is known about the exact steps in triad biogenesis. During development, SR is believed to originate from the rough endoplasmic reticulum [3], while the origin of the T-tubules has been debated for decades [2]. Evidence has suggested that T-tubule formation starts at membrane caveolae [4–6], and continues by repetitive caveolar invaginations at the plasma membrane in the absence of caveolae fission [7]. Alternatively, T-tubule formation could be mediated by a process involving exocytosis of membranes that are not incorporated into the plasma membrane but instead internalized into tubular structures in the cytoplasm to form the T system [3].

Defects at the triad underlie a wide range of human muscle disorders including centronuclear myopathies (CNMs), a clinically and genetically heterogeneous group of rare congenital myopathies [1]. Classical features of CNM include an increased proportion of centralized nuclei on muscle biopsies, where the name of CNM derives from, and abnormalities in triad structure and EC coupling. CNM patients show hypotonia (decreased muscle tone) and muscle weakness that can range from mild to severe and often includes facial and eye movement muscles, and symptoms are often present at birth in the severe forms. CNMs can be attributed to autosomal dominant (ADCNM) mutations in *DNM2* encoding dynamin 2 (19p13.2) [8, 9], X-linked (XLCNM or XLMTM) recessive mutations in *MTM1* encoding myotubularin (Xq28; a lipid phosphatase that acts on phosphoinositides) [10], or autosomal recessive (ARCNM) mutations in *BIN1* encoding amphiphysin-2 (2q14.3; a BAR domain-containing, membrane-tubulating protein) [11], *RYR1* encoding the skeletal muscle ryanodine receptor 1 (19q13.2; an SR localized calcium channel) [12], or *TTN* encoding titin (2p31.2, a giant protein at the sarcomere with many functions including serving as a molecular spring) [13]. XLCNM occurs at a rate of 1 in 50,000 male births, while epidemiological data for the autosomal forms are currently not established. A recent study using an integrated modeling methodology predicted the incidence of non–X-linked CNM to be approximately 7 per million [14]. ADCNM affects both males and females equally with an overall decreased disease severity as compared with that of XLCNM.

Disease mechanisms underlying CNM remain to be clarified. The defective proteins implicated in CNMs to date either directly participate in EC coupling (i.e., *RYR1*) or are involved in various aspects of membrane trafficking events important for triad formation and/or maintenance (i.e., *DNM2*, *MTM1*, and *BIN1*), highlighting the importance of these cellular processes in muscle function.

DNM2 has emerged as a key player in CNM pathogenesis. DNM2 belongs to the dynamin family of large GTPases that mediate membrane fission during multiple cellular processes including endocytosis and organelle division/fusion [15]: DNM1 (dynamin 1) is expressed in neuronal cells, DNM2 is ubiquitously expressed, and DNM3 (dynamin 3) is localized in the brain, heart, testis, and lungs [16]. In addition to CNM, mutations in *DNM2* also lead to intermediate and axonal forms of Charcot–Marie–Tooth disease (CMT), a disorder of the peripheral nerve. It remains unclear why skeletal muscle and peripheral nerves are the main tissues affected by dominant mutations in the ubiquitously expressed DNM2. ADCNM caused by *DNM2* mutations was first identified as a childhood or adolescence-onset muscle disorder characterized by delayed motor milestones and mild–moderate muscular weakness [8]. However, subsequent studies have identified several *DNM2* mutations as a cause of neonatal-onset severe muscle weakness [9, 17, 18], thus expanding the spectrum of DNM2-related myopathies to include essentially all ages and ranges of severity.

Importantly, in XLCNM and ARCNM mouse models, DNM2 protein levels were elevated [19] and reducing DNM2 protein expression is able to revert the CNM phenotypes in these mice [20–22]. In addition, CNM phenotypes can be induced in miR-133a null mice, a microRNA that targets *DNM2* [23], further demonstrating that DNM2 level is important to healthy muscle. These findings using mouse models emphasize the need to study DNM2-mediated pathways in CNM pathogenesis and to understand the interlay between DNM2 and other disease genes causing ARCNM and XLCNM such as *BIN1* and *MTM1*. The aim of this review is to further the understanding of CNM pathogenesis, by providing mechanistic insights into ADCNM (or DNM2-CNM), followed by discussion of DNM2 as a contributor to both ARCNM and XLCNM, and concluding with addressing DNM2 modulation as a potential therapeutic target for human disease.

## DNM2 Structure and Membrane Fission Activity

**Fig. 2:**
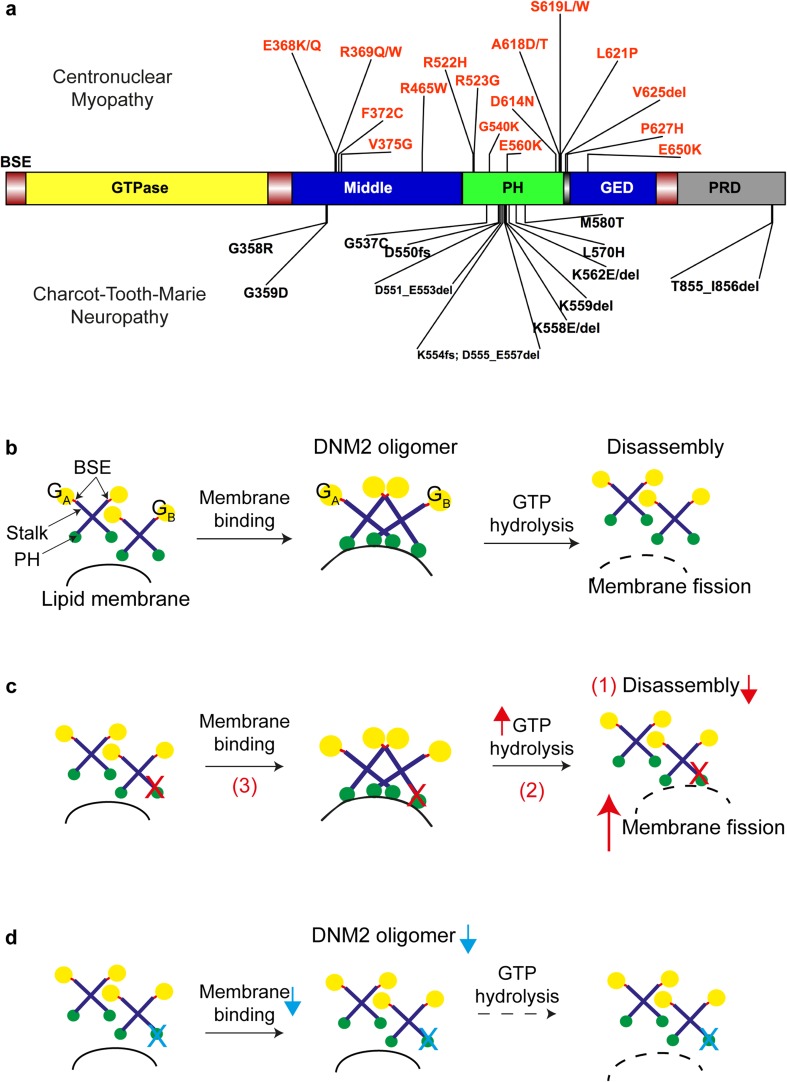
Dynamin domain organization, DNM2 disease mutations, and its oligomerization/disassembly process in healthy and disease states. (a) Dynamin consists of 5 domains: an N-terminal GTPase or G domain (*yellow*), a middle domain (*blue*), a pleckstrin homology (PH) domain (*green*), a GTPase effector domain (GED, *blue*), and a C-terminal proline/arginine-rich domain (PRD, *gray*). The bundle signaling element (BSE, *bright red*) is located at the N- and C-termini of the G domain and at the C-terminus of GED. Mutations in DNM2 cluster at the stalk (middle domain and GED, *blue*) and PH domain, and cause either centronuclear myopathy (*top*, *light red*) or Charcot–Marie–Tooth neuropathy (*bottom*). Mutations that cause early-onset CNM are located at or nearby the PH-GED linker region, i.e., A618D/T, S619L/W, L621P, V625del, and P627H. (b) In the healthy state, dynamin first forms dimers and then further oligomerizes upon lipid-membrane binding. G_A_ and G_B_ are used to label adjacent dimers in a DNM2 polymer. Temporal and spatial control of dynamin oligomerization is mediated by the PH region. The PH domain autoinhibits the stalk to ensure lipid binding occurs before oligomerization. Membrane fission and DNM2 disassembly are then mediated by GTP hydrolysis. (c) In CNM models that carry DNM2 mutations at the stalk/PH region (*red crosses*), the observed elevated GTPase activity as well as membrane fission (i.e., DNM2 hyperactivity) might be a result of a dysregulated assembly/disassembly process indicated by (*1*) the formation of more stable oligomers upon lipid binding (e.g., R465W, A168T), i.e., disrupted disassembly; (*2*) conducting GTPase activity and membrane fission at a higher rate (lipid-sensitized) (e.g., A618T), i.e., more efficient GTP hydrolysis; and/or (*3*) reaching full GTPase activity without lipid binding (lipid-uncoupled) (e.g., S619L/W), i.e., loss of spatial control over oligomerization. (d) In contrast, CMT models that carry mutations mainly at the lipid-binding PH domain (*light blue crosses*) have shown impaired lipid binding. As lipid binding is required for oligomerization, predictably less DNM2 oligomers will be present at the membrane and thus the mildly impaired membrane fission activity (i.e., DNM2 hypoactivity) observed in CMT models. Adobe Illustrator CS6 and IBS (Illustrator for Biological Sequences) were used to generate this diagram

Dynamins are a family of large, multidomain GTPases that can assemble into helical polymers that wrap around the neck of a budding vesicle [15]. Dynamins consist of 5 domains (Fig. 2a): an N-terminal GTPase domain, a middle domain with putative actin-binding motif, a pleckstrin homology (PH) domain that binds to phosphoinositides, a GTPase effector domain (GED) that regulates GTPase activity, and a C-terminal proline/arginine-rich domain (PRD) that binds SH3-domain-containing partners [15, 24]. The middle domain together with GED forms a “stalk” that mediates dynamin dimerization. The N- and C-terminal helices of the GTPase domain and the C-terminal helix of the GED together form the 3-helix bundle signaling element (BSE), a flexible hinge connecting the GTPase domain and the stalk. BSE is able to sense and transmit the conformational changes associated with dynamin assembly to the GTPase domain [25]. Dynamin first forms anti-parallel dimers [15] that can assemble into oligomers upon lipid-membrane binding (Fig. 2b). The PH domain plays an autoinhibitory role in dynamin assembly by ensuring membrane binding occurs prior to the formation of higher-order oligomers [26, 27]. Membrane fission and oligomer disassembly, the order of which remains a debate [15], are then mediated by nucleotide-driven conformational changes [15, 28] and GTP hydrolysis [25, 29–31].

Dynamin was the first protein shown to catalyze membrane fission [32]. Dynamin-mediated membrane fission during endocytic vesicle release has been intensively studied and well established [33, 34]. Although most of the studies have focused on DNM1, the DNM proteins share high sequence similarity with each other, and are thus likely to have evolved to similarly regulate endocytosis in different tissue types [35]. It was first revealed in the *Drosophila shibire* mutants that mutations in dynamin result in accumulation of endocytic profiles mainly at the membrane neck [36]. Dynamin is subsequently shown to regulate both clathrin-coated pit (CCP) maturation and vesicle release during both clathrin-mediated endocytosis (CME) [37] and caveolae-mediated endocytosis [38–40].

## *DNM2* Mutations and Their Effects on Protein Localization and Activity

*DNM2* mutations cause either CNM or CMT, with no reports of any single *DNM2* mutation causing both diseases (Fig. 2a) [41, 42]. A homozygous mutation in *DNM2* causes a lethal congenital syndrome (reported in 1 family) [43]. Pathogenic mutations are often missense or in-frame indels, with the majority clustering to the PH domain and interface of PH and stalk domains. At present, 60 variants have been reported in the Leiden Open Variation Database. The recurrent p.R465W mutation is the most common, and accounts for approximately 25% of affected families, whereas the p.E368K and p.R369W mutations, along with those in residues 618 and 619, are found in approximately 20%, 10%, and 15% of families, respectively.

Cytosolic DNM2 accumulation has been reported in both *in vitro* [34, 44–49] and *in vivo* [19, 50–53] models expressing DNM2 mutations. Normally, dynamin is localized at the plasma membrane, at the perinuclear region, and potentially at the endosomal compartment and the Golgi network [45, 49]. DNM2 is also localized to tissue-specific structures in isolated fibers of mouse muscle, such as the postsynaptic neuromuscular junction [51] and regions nearby sarcomeric Z-disks in proximity to the triad [19, 51]. In transfected mammalian fibroblasts, while CMT-DNM2 mutant proteins show a comparable pattern to the wild type [45, 49], CNM mutant DNM2s no longer colocalize with a Golgi marker (R465W, E368K) [45], and are present in enlarged cytosolic punctate (P627H, S619L/W, R522H, R465W) [49]. In muscle fibers isolated from a heterozygous mouse model expressing DNM2^R465W^ (HTZ), large DNM2 accumulations containing the membrane-repairing protein dysferlin can be observed at the central region of the fiber [51]. Cytosolic DNM2 accumulation could also be observed in transfected mammalian myoblasts (R369W, R465W, and R522H) [53], in the body wall muscle of *Drosophila* larvae stably expressing DNM2 mutant proteins (R465W, A618T, and S619L) [52], and in muscle biopsies of CNM patients (D614N) [46]. Although CMT mutations also lead to aggregates, they appear to be concentrated at the perinuclear region and colocalized with a Golgi marker (DNM2^551∆3^), or partially colocalized with clathrin (DNM2^553X^) in the cytosol [54]. As DNM2 mutations have not been shown to cause protein misfolding [48], the susceptibility of DNM2 mutations to aggregation is possibly due to dysregulated DNM2 oligomerization (normal process as shown in Fig. 2b). Notably, CNM mutations have been shown to elevate GTPase and membrane fission activities [26, 52] without affecting lipid/phosphoinositide binding [44]. This hyperactivity of membrane fission could be a result of the dysregulated oligomerization/disassembly process (Fig. 2c), because 1) CNM mutant proteins (E368K, R369W, R465W, and A618T) can form more stable oligomers that are significantly resistant to disassembly [26, 47], 2) CNM mutant (e.g., A618T) proteins can conduct lipid-stimulated GTPase activity and membrane fission at a higher rate (“lipid-sensitized”) [44], and/or 3) CNM mutant proteins (e.g., S619L/W and V625del) can reach full GTPase activity without lipid binding (“lipid-uncoupled”) [44], i.e., loss of controlled oligomerization. In contrast, CMT mutations could impair lipid binding as demonstrated by reduced *in vitro* liposome cosedimentation [48, 54] and as suggested by their locations within the lipid-binding PH domain [54] (Fig. 2d). Notably, distinct differences in binding to high-curvature membranes were observed between CMT mutations K562E (not binding) and G537C (binding) [52]. This suggests K562E causes membrane-binding defects, while G537C is defective in curvature generation. CMT mutations also mildly reduce membrane fission activity, suggesting that they could cause hypoactivity of the protein [52].

## DNM2-CNM Disease Mechanisms

### Overview

**Fig. 3:**
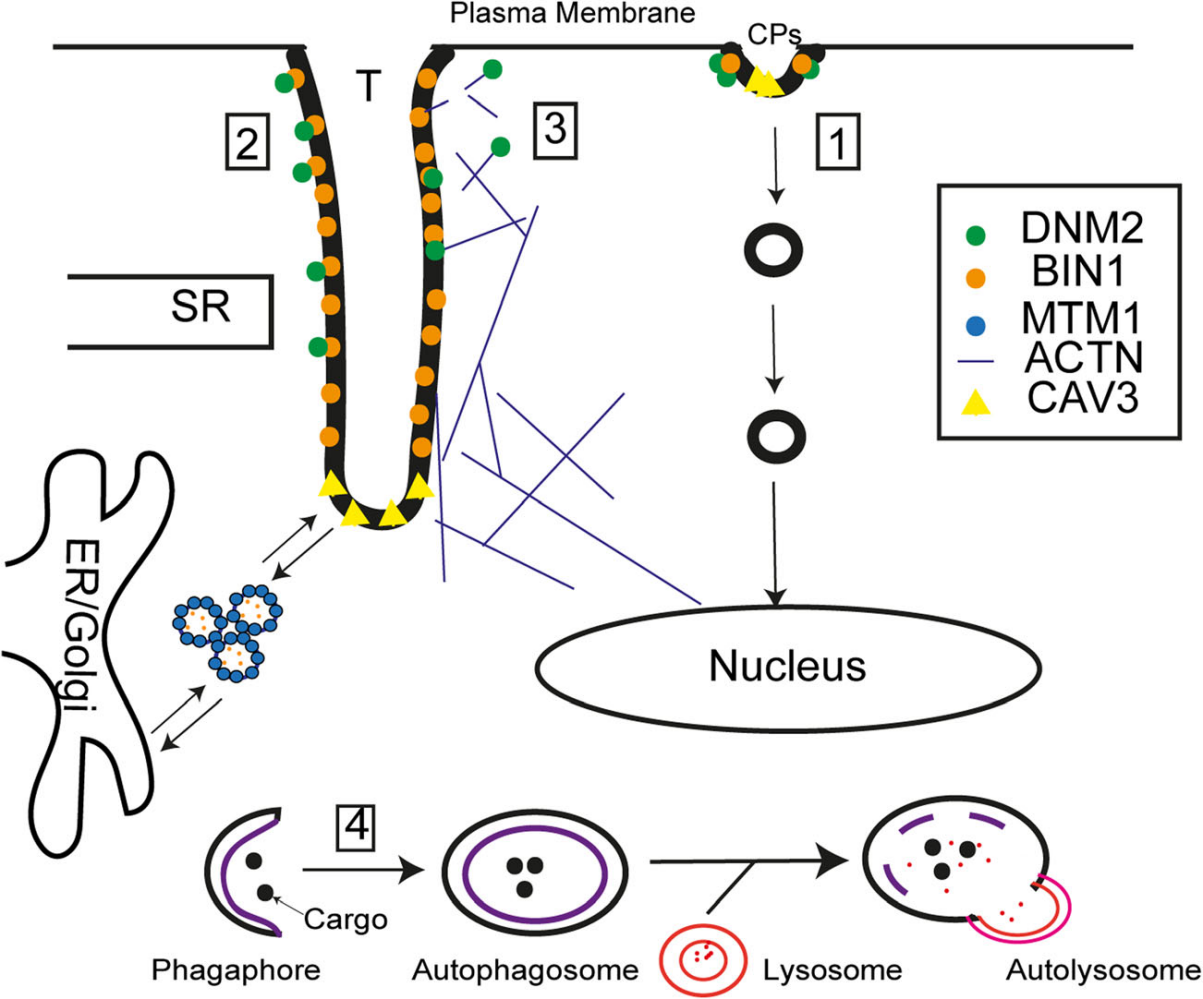
Potential membrane trafficking events disrupted in DNM2 centronuclear myopathy. (1) DNM2 (*green dots*) regulates membrane fission during endocytic vesicle release by binding around the neck of either clathrin- or caveolae-coated pits. Impaired endocytosis has been observed in cells expressing CNM-DNM2. (2) The triad [T-tubules (T) and sarcoplasmic reticulum (SR)] is a system of membrane invaginations that regulate EC coupling in muscle. DNM2 is localized in proximity to the triad, while its exact subcellular localization is unknown. However, DNM2 can bind to BIN1 (*orange dots*) at the T-tubules, another CNM protein that regulates tubulogenesis. BIN1 is localized to the tubular portion of T-tubules, while CAV3 (*yellow triangles*) is localized to the vesicular ends. While the steps of T-tubule biogenesis remain a debate, the interplay between MTM1 (*red dots*), BIN1, and DNM2 has been shown to be important for triad biogenesis and/or maintenance. Hyperactivity of DNM2 (e.g., caused by some CNM mutations) can lead to severe fragmentation of T-tubules. (3) DNM2 can either directly bind to cytoskeletal actin (*blue lines*) to promote actin polymerization, or regulates actin dynamics via binding to BIN1, which binds to cytoskeletal actin via its BAR domain. Actin dynamics is involved in tubulogenesis and myonuclei positioning in cells, and can be disturbed by some CNM mutations. (4) DNM2 can also regulate the maturation of phagophore to autophagosome during autophagy by retrieving Atg9 after Atg9-regulated membrane addition onto phagophores. This may explain the autophagic blockage observed in some CNM models. Adobe Illustrator CS6 was used to create this diagram

As discussed earlier, DNM2 is well known for its role in mediating membrane fission during endocytic vesicle release. It is thus intuitive to hypothesize dysregulated endocytosis as the underlying pathomechanism of DNM2 diseases. Indeed, some CMT mutations can impair CME efficiency [51, 55, 56], but not all CMT mutations lead to significant reduction in CME efficiency (e.g., 551∆3) [57], possibly due to mislocalization of mutated DNM2 from endocytic compartments. Similarly, it remains controversial whether CME efficiency is impaired by CNM mutations due to discrepancies observed between *in vitro* overexpression systems and, more importantly, between *in vitro* systems and CNM patient fibroblasts. Depending on the cell type, CNM mutations displayed impaired CME in transfected COS-7 cells (R465W, V625del, and E650K) [55], COS-1 cells (R465W and S619L) [49], and embryonic fibroblasts from the lethal homozygous DNM2^R465W^ mouse model (HMZ) [51]. In contrast, wild-type comparable CME efficiency was observed in transfected mouse motor neuronal cells, HeLa cells (E368K/Q, E369W, R465W, E560K, A618T, S619L/W, V625del, and E650K) [56], C2C12 myoblasts (R465W, A618T, S619L) [52], epithelial cells (S619L) [27], and patient fibroblasts (R465W, S619L) [49]. To overcome the possibility of inducing overexpression phenotypes and better mimic patient conditions, Liu et al. [45] utilized endogenous Dyn2 knock-out mouse fibroblasts for heterozygous expression of CNM mutants (E368K and R465W), which also detected no changes in CME. Notably, CNM-DNM2 might not participate in endocytosis as Srinivasan et al. [27] observed inefficient recruitment of DNM2^S619L^-eGFP to the plasma membrane. Moreover, the hypothesis of impaired endocytosis cannot explain the muscle specificity of ADCNM. It is therefore important to examine nonendocytic pathways that are likely at play in muscle cells of ADCNM. Although the role of DNM2 in muscle remains largely unknown, *in vitro* and *in vivo* models of DNM2-CNM have suggested dysregulation of T-tubule biogenesis/maintenance [52, 58], cytoskeletal remodeling [53], and autophagy [50] as potential pathogenic mechanisms underlying CNM [41]. This section discusses the role of DNM2 in these pathways and the possibility of its disruption as a pathogenic mechanism related to CNM (Fig. 3).

### Disrupted T-Tubule Biogenesis and/or Maintenance

The triad (T-tubules and SR) is a cellular structure unique to skeletal muscle cells, and abnormal triads are a key feature in CNM patients (as mentioned in “Introduction”). *Drosophila* transgenic lines overexpressing muscle-specific wild type or CNM-causing DNM2 (WT, R465W, A618T, and S619L), but not CMT-causing DNM2 (G537R), led to cytoplasmic punctate distribution of a T-tubule marker, indicating that the hyperactive DNM2s can cause severe fragmentation of the T-tubules in muscles [52]. Similarly in zebrafish, Dnm2a/2b are required for normal muscle development [59], and overexpressing a human CNM mutation (i.e., S619L) leads to disorganized triads with extensive swelling and vacuolization [58]. The detrimental effect of CNM mutations on tubulation is also demonstrated in COS-7, where BIN1-inducing tubulation is completely disrupted by ectopic expression of DNM2^S619L^ but not DNM2^wild type^ [58]. These findings indicate the CNM-DNM2 mutations may primarily affect the triad structure in skeletal muscle by acting as an antagonist to tubulogenesis and/or tubule maintenance.

Several membrane scaffolding proteins, like caveolin-3, junctophilin-2, and BIN1, have been implicated in T-tubule biogenesis [60]. Follow-up studies using transgenic mice with deletion of these genes individually reveal that the primary T-tubule invaginations still exist, indicating that no single protein is required for tubulogenesis [60]. Immunogold staining on muscle EM section confirmed the localization of BIN1 along the tubular portion of the T-tubules, and the caveolar protein caveolin-3 at the vesicular portion [6]. Immunofluorescence staining of wild-type mouse skeletal muscle, however, reveals colocalization of DNM2 with α-actinin at the sarcomeric Z-disk but not with the DHPR receptor on T-tubules [61]. This suggests that DNM2 may only be in spatial proximity but not localized to the T-tubules, challenging that DNM2 could directly regulate T-tubule biogenesis.

A growing body of evidence suggests that DNM2 may also play a role in membrane tubulation by virtue of its speculated interaction with *BIN1* [11, 62–64]. Indeed, in the absence of *MTM1*, BIN1 expression is disorganized at the mature T-tubule [63], suggesting that defective membrane tubulation may underlie the structural disorganization of the triad in myopathic muscle. In light of its well-established role as a sensor and inducer of membrane curvature, recent studies suggest that BIN1 mediates T-tubule biogenesis and is capable of recruiting DNM2 to endocytic sites via its C-terminal SH3 domain [65, 66]. Although DNM2 partially colocalizes with BIN1-labeled tubular structures in C2C12 myotubes [61], and caveolin-3-labeled T-tubule-adjacent structures [61], whether DNM2 and BIN1 directly interact in the muscle is not clear; nor is it completely understood whether either protein functions to regulate the other within skeletal muscle. This is in contrast to MTM1, which colocalizes with BIN1 at the T-tubule and, when bound, has been shown to enhance BIN1-mediated membrane tubulation *in vitro* [63]. Whether the involvement of MTM1 and/or speculated role of DNM2 in T-tubule formation contributes towards triad dysregulation is unknown and merits further investigation. In addition, it is still unclear how disrupted T-tubules may cause the centralized myonuclei positioning phenotype seen in CNM patients, or whether this is a separate process. Although impaired myofibril contraction can lead to misplaced myonuclei [67], not all triadopathies show centralized nuclei [1], suggesting additional pathway(s) besides T-tubule biogenesis/maintenance may also play a role in CNM pathogenesis.

### Disrupted Actin Dynamics

Accumulating evidence has suggested that cytoskeletal actin and its associated interactions play an important role in CNM pathogenesis. Disrupting actin polymerization is able to affect T-tubule biogenesis in cultured cardiomyocytes [60] as well as myonuclei positioning in muscle cells [67]. In HTZ mice, the heterozygous *DNM2* mutation leads to altered actin organization and reduced actin polymerization [53]. N-WASP, the actin nucleation-promoting factor, is mislocalized from the triad to accumulate around the centrally located nuclei in BIN1-CNM patients [68]. Moreover, the skeletal muscle-specific ablation of γ-actin in mice progressively leads to CNM-like phenotypes including centralized myonuclei and triad abnormality [69], providing a direct link between disrupted actin network and CNM pathogenesis. However, how DNM2 regulates actin dynamics in the context of membrane trafficking remains unclear. Gu et al. [24] identified actin-binding sites at the DNM2 stalk region, and mammalian myoblasts transfected with DNM2 carrying mutations in the region reduced *de novo* actin filament formation [53], demonstrating that CNM mutations could directly affect actin polymerization in muscle cells. During membrane tubulation, BIN1 remodels actin filaments via its BAR domain [70], and an inhibitor to actin polymerization was shown having opposite effects to DNM2 overexpression on BIN-inducing tubular invagination [71]. These findings suggest that DNM2 could also indirectly regulate actin dynamics through BIN1 interaction.

### Impaired Autophagy

Besides disrupted actin network, impaired autophagy has also been reported in mouse models of ADCNM [50, 51]. Autophagy consists of highly regulated steps: sequestration or formation of autophagosome, trafficking, and fusion with lysosomes for degradation [72]. Newly added autophagic membranes are derived from other organelles through the formation of vesicles carrying Atg9 (autophagy-associated protein 9). Dnm2 is shown by a recent study to be localized to autophagic membranes, and to regulate Atg9 retrieval from early autophagosomes [73], suggesting that DNM2 may play a role in autophagosome maturation. It still remains an open question whether impaired autophagy could be the pathogenic mechanism underlying CNM. Indeed, aberrant autophagy has been reported in different CNMs including XLCNM [74] and ARCNM [75]. Autophagy is also involved in fiber type-specific atrophy [76], which may explain the type I predominance and hypotrophy observed in many DNM2-CNM patients. However, DNM2-CNM patients do not show typical autophagy phenotypes such as those seen in autophagic vacuolar myopathy (AVM) [77], a neuromuscular condition characterized by increased muscle glycogen and intracytoplasmic vacuoles, and the AVM patients with disrupted autophagy do not exhibit CNM-like triad and myonuclei phenotypes.

### Summary

In summary, (presumably) hyperactive *DNM2* mutations promote defects in multiple cellular pathways, including T-tubule biogenesis, actin dynamics, and autophagy. While these mechanisms are not mutually exclusive to each other, further investigation is required to examine whether the CNM mutations affect 1 principal pathway that leads to changes in others or multiple pathways simultaneously.

## DNM2 Modulation as a Therapeutic Target for Muscle Disease

In the absence of any proven disease-modifying therapeutic candidates for this devastating group of muscle diseases, many groups have sought to explore whether phenotypic overlap amongst CNMs arises by way of a unifying pathomechanism. In support of this, Cowling et al. [19] recently identified *DNM2* as a novel genetic modifier of *MTM1* [78]. Using an *Mtm1* knock-out (KO) mouse model, Cowling and colleagues [19] demonstrated that DNM2 protein expression was significantly elevated in the tibialis anterior of the *Mtm1* KO mouse. This was further substantiated by their observation that XLCNM patient fibroblasts also overexpressed DNM2 at the protein level [19, 78], suggesting that elevated DNM2 levels may act as a pathogenic contributor in XLCNM. Homozygous deletion of *Dnm2* (Dnm2^−/−^) is embryonically lethal in mice, whereas heterozygous *Dnm2*^*+*/−^ mice (expressing half the normal protein level of DNM2) are phenotypically normal. Taking advantage of this, Cowling and colleagues [19] reduced DNM2 protein levels by approximately half in *Mtm1* KO mice by generating *Mtm1* KO mice that were heterozygous for *Dnm2* (*Dnm2*^*−/+*^*Mtm1*^*−/−*^). Remarkably, *Dnm2* reduction was sufficient to improve survival and to restore aspects of muscle function and triad structure of *Mtm1* KO mice [19].

This study was important because it was the first of its kind to demonstrate the therapeutic potential of DNM2 reduction. In this way, evidence gleaned from this study lent further support towards a hypothesis that CNM phenotypes arise as a consequence of DNM2 hyperactivity. This concept is additionally supported by the fact that overexpression of wild-type DNM2 in mice [61], or deletion of a micro RNA that negatively regulates DNM2 levels [23], results in a CNM-like phenotype. Taken with the understanding of DNM2 mutations, and the fact that reducing DNM2 can rescue CNM phenotypes in non-DNM2 models, there emerges the idea that DNM2 overexpression is both necessary and sufficient to cause most/all aspects of CNM pathology.

Following the discovery that reducing DNM2 can improve the phenotype of the *Mtm1* KO mouse, efforts were made to reduce DNM2 using clinically relevant strategies. Using both anti-sense oligonucleotides (ASOs) and intramuscular injection of AAV-shRNA against *Dnm2 in vitro* and *in vivo*, Tasfaout et al. [21, 22] successfully downregulated DNM2 in the *Mtm1* KO background. Both strategies resulted in long-term reduction of DNM2 protein levels and prevented the development of XLCNM phenotypes in *Mtm1* KO mice. Indeed, both ASO- and AAV-shRNA-mediated DNM2 knockdown restored muscle force, mass, and histology and prevented the overall development of muscle-specific phenotypes.

Similarly, a study conducted by Cowling et al. in 2017 [20] was the first to demonstrate the therapeutic benefit of DNM2 downregulation in BIN-1-related CNM (i.e., ARCNM). Given that the complete loss of *Bin1* (*Bin1*^*−/−*^) is embryonically lethal, Cowling and colleagues sought to rescue this lethality by genetically reducing DNM2 levels in mice homozygous for *Bin1*. Not only was this rationale based on insights gleaned from their previous studies in *Mtm1* KO mice, but it also arose from the desire to decipher the molecular interplay between DNM2 and BIN1. In like manner to conclusions from their previous study, *Bin1*^−/−^ mice heterozygous for *Dnm2* (*Bin1*^*−/−*^*Dnm2*^*+/−*^) survived for up to 18 months and exhibited normal muscle histology and ultrastructure [20]. Additional *in vitro* analyses demonstrated that BIN1 acts to negatively regulate the GTPase activity of an “immature” muscle-specific isoform of DNM2 during skeletal muscle development and maturation. This was further supported by the loss of this regulation, and the predominant expression of an “adult” isoform of DNM2, during skeletal muscle maintenance in the later stages of muscle fiber development [20]. These studies provide evidence in support of an important interplay between BIN1 and DNM2 during myogenesis and within the context of BIN1-related CNM. Ultimately, these findings are important because they set a precedent for studying shared regulatory mechanisms between CNM-associated genes in specific tissues and stages of development in order to further understand CNM pathophysiology.

Lastly, in a model of DNM2-related CNM, Trochet et al. [79] successfully reduced mutant DNM2 protein and mRNA levels in murine and patient fibroblast models of ADCNM using allele-specific silencing RNA (siRNA) against the ADCNM-associated p.R465W mutation. This reduction was sufficient to achieve restoration of muscle function in a transgenic knock-in mouse model with the same mutation.

Taken together, these studies showcase DNM2 modulation as a potential therapeutic approach for both autosomal and X-linked forms of CNM, and suggest that a unifying disease mechanism exists between these diseases that may be amenable to therapeutic intervention. Furthermore, evidence gleaned from these studies is in agreement with the hypothesis that DNM2 hyperactivity is a likely pathogenic mechanism in CNMs and suggests that the molecular cause(s) underlying DNM2 dysregulation in ADCNM and XLCNM are similar. Indeed, it is possible that DNM2 overexpression and subsequent aberrant molecular activity drive the development of muscular abnormalities observed in both autosomal and X-linked forms of CNM.

## Concluding Remarks

DNM2 is a membrane fission protein well known for its role in regulating endocytic vesicle release, and has emerged as a crucial player in the pathogenesis of centronuclear myopathy. While the muscle-specific role of DNM2 remains largely unknown, DNM2 closely interacts with BIN1, which together with MTM1 participates in membrane trafficking in muscle cells. Muscle membrane trafficking involves multiple cellular pathways that are individually tightly regulated, and also closely associated with each other, including T-tubule biogenesis, actin dynamics, and autophagy. Studying the role of DNM2 in ADCNM, XLCNM, and ARCNM will advance understanding of many unsolved questions regarding muscle development and maintenance, and help develop therapeutic approaches to treat these diseases.

## Electronic Supplementary Material

ESM 1 (PDF 1225 kb)
